# Molecular characterization of NDM-1-producing carbapenem-resistant *E. cloacae* complex from a tertiary hospital in Chongqing, China

**DOI:** 10.3389/fcimb.2022.935165

**Published:** 2022-08-08

**Authors:** Kewang Hu, Jisheng Zhang, Jingbo Zou, Lingyi Zeng, Jie Li, Jianmin Wang, Wenzhang Long, Xiaoli Zhang

**Affiliations:** ^1^ Department of Microbiology, Yongchuan Hospital of Chongqing Medical University, Chongqing, China; ^2^ Department of Microbiology, Affiliated Hangzhou Xixi Hospital, Zhejiang University School of Medicine, Hangzhou, China; ^3^ Department of Microbiology, Yongchuan District Center for Disease Control and Prevention of Chongqing, Chongqing, China; ^4^ Department of Molecular biology, Jiaxing Maternal and Child Health Hospital, Jiaxing, China

**Keywords:** carbapenem-resistant, *Enterobacter cloacae* complex, NDM-1, mcr-9, plasmids

## Abstract

**Background:**

The aim of this study was to clarify the molecular characterization of NDM-1-producing carbapenem-resistant *Enterobacter cloacae *complex (CREL) at a teaching hospital in Chongqing, China.

**Methods:**

Antimicrobial susceptibility and resistance genes were analyzed. Epidemiological relationship was analyzed by pulsed-field gel electrophoresis (PFGE) and multilocus sequence typing (MLST). Conjugation experiments were performed to determine the transferability of plasmids. Whole-genome sequencing (WGS) of strains was implemented, and the genetic environment of the *bla*_NDM-1_- and *mcr-9*-carrying plasmids was analyzed.

**Results:**

A total of 10 *bla*_NDM-1_-positive CREL isolates were identified. All isolates harbored multiple resistance genes. ECL68 and ECL78 co-produce *bla*_NDM-1_ and *mcr-9*. Among the four different sequence types (STs) detected, ST1466 was assigned as a novel ST. Six isolates exhibited highly similar PFGE patterns. Conjugation assay proved that all plasmids containing *bla*_NDM-1_ or *mcr-9* could be transferred to the recipient *Escherichia coli*. WGS indicated that *bla*_NDM-1_ genes were carried by diverse plasmids, including IncHI2/IncN, IncX3, and one unclassified plasmid type. The backbone structure of these plasmids is involved in replication initiation (*repAB*), partitioning (*parABM*), and conjugation/type IV secretion (*tra/virB*). Analysis of the genetic environment showed that *bla*_NDM-1_ in three plasmids exhibited a highly similar structure to protype Tn*125*. Co-existence of *bla*_NDM-1_ and the colistin resistance gene *mcr-9* was detected in the two isolates, ECL68 and ECL78. In ECL68, *bla*_NDM-1_ and *mcr-9* were present on the same plasmid while located in two separate plasmids in ECL78. The genetic environment of *mcr-9* was organized as IS*26-wbuC-mcr-9-*IS*903-pcoS-pcoE-rcnA-rcnR*, and the two-component system encoding genes *qseC* and *qseB* was not found in two plasmids, which could explain *mcr-9*-harboring strains’ colistin susceptibility.

**Conclusions:**

We first report a nosocomial outbreak of NDM-1-producing *E. cloacae complex* ST177 in China. Conjugative plasmids contributed to the horizontal transfer of antibiotic resistance genes. The prevalence and even coexistence of *bla*_NDM-1_ and *mcr-9* may further threaten public health. Our results highlight further surveillance for *bla*_NDM-1_, and *mcr-9* is essential to prevent its dissemination.

## Introduction

In recent years, the prevalence of carbapenem-resistant *Enterobacteriaceae* (CRE) has become the major reason for clinical anti-infective treatment failure and posed a serious threat to clinical management (Perez and Bonomo, 2019). Infections caused by CRE have a higher chance of having severe clinical consequences compared with other pathogens (Falagas et al., 2014). The main mechanism of carbapenem resistance includes production of carbapenemases, active efflux of bacteria, and mutation of outer membrane proteins (Majewski et al., 2016).

*Enterobacter cloacae* complex (ECL), a species of the *Enterobacteriaceae* family, is inherently resistant to first- and second-generation cephalosporin due to chromosomally mediated AmpC β-lactamase (Jacoby, 2009). The ECL bacteria are related to a series of nosocomial infections including pneumonia, urinary tract infections, and septicemia. The overuse of broad-spectrum antibiotics resulted in the emergence of multidrug-resistant and even carbapenem-resistant *E. cloacae* (CREL) around the world. According to CHINET surveillance data, the detected rate of CREL ranked third among all *Enterobacteriaceae*, just after carbapenem-resistant *Klebsiella pneumoniae* and *Escherichia coli* (Lee et al., 2017; Annavajhala et al., 2019; Tetsuka et al., 2019).

In 2009, New Delhi metallo-β-lactamase-1 (*NDM-1*), also known as Ambler class B metallo-β-lactamase, was first reported in a clinic *K. pneumoniae* in India and commonly located in conjugative plasmids (Yong et al., 2009). In the following few years, clinical strains carrying the *bla*_NDM-1_ appeared in more than 50 countries (Kumarasamy et al., 2010; Walsh, 2010; Nordmann et al., 2011). This spread and prevalence across species indicates that *bla*_NDM-1_ is seriously threatening public health in a new way (Boyd et al., 2020). However, there have been little epidemiological data on NDM-1-producing CREL in our region. Thus, our study aimed to investigate the prevalence of CREL and molecular characteristics of the *bla*_NDM-1_-carrying plasmids in Chongqing.

## Materials and methods

### Collection and identification of bacterial isolates

From 30 June 2018 to 30 June 2020, 10 non-duplicated NDM-1*-*producing CREL isolates were collected from the Yongchuan Hospital Affiliated of Chongqing Medical University. Bacterial species identification was performed by using the VITEK2 compact automated system (bioMerieux, France). The *Enterobacter cloacae* complex was further identified to the subspecies level by *16S* rRNA as described previously (Mezzatesta et al., 2012). All isolates were preserved at −80°C until further study.

### Antimicrobial susceptibility testing

Initial antibiotic susceptibility was analyzed by using the VITEK2 compact system. MICs of imipenem (IPM), meropenem (MEM), levofloxacin (LEV), amikacin (AMK), tigecycline (TGC), and polymyxin B (PB) were evaluated using the broth microdilution method. The results were interpreted according to the interpretive criteria from CLSI 2020. The tigecycline was interpreted according to the European Committee on Antimicrobial Susceptibility Testing (EUCAST) guidelines. *E. coli* ATCC 25922 served as the quality control strain for susceptibility testing.

### Phenotype testing and detection of antibiotic-resistant genes

Phenotypic screening was based on the CLSI 2020 guidelines. The modified carbapenem inactivation method (mCIM) and the EDTA-modified carbapenem inactivation method (eCIM) were used to screen carbapenemase production. The PCR was performed to detect the presence of carbapenemase-related genes (*bla*_KPC_, *bla*_NDM_, *bla*_VIM_, *bla*_IMP_, and *bla*_OXA−48_). Moreover, ESBLs, AmpC, and resistance genes for fluoroquinolones were determined by using primers as described previously (Gong et al., 2018).

### Multilocus sequence typing and pulsed-field gel electrophoresis

Multilocus sequence typing (MLST) was used to detect the sequence types. Seven housekeeping genes (*dnaA*, *fusA*, *gyrB*, *leuS*, *pyrG*, *rplB*, and *rpoB*) were submitted to the MLST database (https://pubmlst.org/ecloacae/). New allele and profile were approved by the MLST website. Molecular phylogenetic analyses were carried out on MEGA.X software. Moreover, pulsed-field gel electrophoresis (PFGE) was used to further determine the genetic relatedness based on the protocol (Tenover et al., 1995). The DNA patterns were analyzed by BioNumerics software v6.6 (Applied Maths, Kortrijk, Belgium). Isolates were allocated into genetic similarity clusters using an 80% cutoff value (Zhao et al., 2020).

### Conjugation experiment

The transferability of resistance gene was determined using a conjugation experiment. As previously described (Wang et al., 2015; Zeng et al., 2021), the conjugation test was performed by the membrane bonding method using NDM-1-producing CREL as the donor and rifampin-resistant *E. coli EC600* as the recipient. Briefly, both the donor and the recipient strains were mixed on Luria-Bertani agar at a ratio of 1:2, and the mixtures were incubated at 37°C overnight. Transconjugants were selected on Mueller-Hinton agar plates supplemented with a combination of 1 µg/ml MEM and 600 µg/ml rifampicin. The resistance gene and MIC in the tranconjugants were confirmed by PCR and antimicrobial susceptibility testing.

### Whole-genome sequencing and data analysis

Genomic DNA of CREL 68, 72, 78, and 112 isolates were prepared using the MagAttract HMW DNA Kit (Qiagen, Hilden, Germany) and subjected to whole-genome sequencing (WGS) using the HiSeq 2000™ platform (Illumina Inc., San Diego, CA, USA) with 2 × 100-bp paired-end reads and to long-read high-throughput sequencing (LRS) on a MinION platform (Oxford Nanopore Technologies, Oxford, UK). The long read generated by MinION was assembled using Canu v. 1.6 and polished with the short reads generated by HiSeq using Pilon v1.22 to obtain the whole genome and complete plasmid sequences. The genomic sequence was annotated using the NCBI Prokaryotic Genome Annotation Pipeline (PGAP) and Glimmer 3.02 (http://www.cbcb.umd.edu/software/glimmer/). Plasmid replicon types were identified using PlasmidFinder (https://cge.cbs.dtu.dk//services/PlasmidFinder/). Antibiotic resistance genes were identified using both the Comprehensive Antibiotic Resistance Database (CARD) and ResFinder database (https://cge.cbs.dtu.dk/services/ResFinder/). Transposon and insertion sequence (IS) elements were identified using the ISfinder database (https://www-is.biotoul.fr/). Linear comparison of sequences was performed using BLAST with the default settings (the nucleotide collection database and the megablast program), and visualized by BRIG (http://brig.sourceforge.net) or Easyfig tools (https://github.com/mjsull/Easyfig) (Zeng et al., 2021).

### Nucleotide sequence accession numbers

The complete sequences of plasmids pNDM-068001, pMDR-072001, pNDM-072002, pMCR-078001, pNDM-078002, and pNDM-011201 were submitted to GenBank under accession numbers MZ156799, MZ156800, MZ156801, MZ156802, MZ156803, and MZ156804, respectively.

## Results

### Characteristics of collected samples

In this study, a total of 10 nonduplicate NDM-1-producing CREL isolates were investigated during the study period. The most common specimens were respiratory tract (8 cases, 80%), followed by urine (1 case, 10%) and bile (1 case, 10%). The majority of patients belonged to the neonatology ward (*n* = 4, 40.0%), followed by the respiratory and critical care medicine ward (*n* = 2, 20.0%), pediatric intensive care unit PICU (*n* = 1, 10.0%), pediatric ward (*n* = 1, 10.0%), respiratory department (*n * = 1, 10.0%), and hepatobiliary surgery (*n* = 1, 10.0%) (**Table 1**).

**Table 1:** Microbiological and molecular characteristics of *Enterobacter cloacae* strains.

Isolate	Species identification	Date of isolation	Ward	Specimen	ST	PFGE pattern
ECL66	*Enterobacter hormaechei*	1 March 2019	PICU	Sputum	177	A
ECL67	*Enterobacter cloacae subsp. cloacae*	15 May 2019	Pediatrics	Sputum	177	B
ECL68	*Enterobacter hormaechei subsp. xiangfangensis*	11 May 2019	Respiratory and critical care medicine	Sputum	93	C
ECL72	*Enterobacter hormaechei*	16 February 2019	Neonatology ward	Sputum	177	A
ECL73	*Enterobacter hormaechei*	5 February 2019	Neonatology ward	Sputum	177	A
ECL75	*Enterobacter hormaechei*	19 December 2018	Respiratory	Urine	177	A
ECL76	*Enterobacter hormaechei*	19 December 2018	Neonatology ward	Sputum	177	A
ECL78	*Enterobacter cloacae complex*	23 September 2018	Hepatobiliary surgery	Bile	1466	E
ECL79	*Enterobacter hormaechei*	12 November 2018	Neonatology ward	Sputum	177	A
ECL112	*Enterobacter hormaechei*	11 June 2020	Respiratory and critical care medicine	Sputum	171	D

### Antimicrobial susceptibility tests

All NDM-1-producing CREL isolates were defined as MDR as they were resistant to three or more classes of antimicrobial agents. Of all the antimicrobials tested, the most susceptible antimicrobial was amikacin (90%), followed by tigecycline (60%), polymyxin B (60%), ciprofloxacin (60%), and levofloxacin (40%). All strains showed high resistance rates to piperacillin-tazobactam (100%), ceftazidime (100%), cefepime (100%), and aztreonam (100%). The antibiotic susceptibility profile is shown in **Table 2**.

**Table 2:** Antibiotic susceptibilities and resistance determinants of *E. cloacae* isolates and their transconjugants (μg/ml).

Isolate	Carbapenemase	ESBL	AmpC	Fluoroquinolone	IMP	MEM	FEP	CAZ	TZP	CIP	ATM	LEV	AMK	PB	TGC
*Enterobacter cloacae complex*															
ECL66	*NDM-1*	*TEM-1*, *SHV-12*	*ACC*	*aac(6′)-Ib-cr*	16	16	≥64	≥64	≥128	0.5	≥64	<0.5	4	2	<0.5
ECL67	*NDM-1*	*TEM-1*, *SHV-12*	*ACC*	*aac(6′)-Ib-cr*	512	32	≥64	≥64	≥128	0.5	≥64	<0.5	2	2	<0.5
ECL68	*NDM-1*	*TEM-1*	*ACC*, *DHA*	*aac(6′)-Ib-cr, qnrS*	32	64	≥64	≥64	≥128	≥4	≥64	64	1	1	2
ECL72	*NDM-1*	*TEM-1*, *SHV-12*	*ACC*	*aac(6′)-Ib-cr*	32	64	≥64	≥64	≥128	≥4	≥64	4	1	2	8
ECL73	*NDM-1*	*TEM-1*, *SHV-12*	*ACC*	*aac(6′)-Ib-cr*	32	64	≥64	≥64	≥128	≥4	≥64	4	1	4	8
ECL75	*NDM-1*	*TEM-1*, *SHV-12*	*ACC*	*aac(6′)-Ib-cr*	128	16	≥64	≥64	≥128	≥4	≥64	<0.5	1	4	1
ECL76	*NDM-1*	*TEM-1*, *SHV-12*	*ACC*	*aac(6′)-Ib-cr*	16	32	≥64	≥64	≥128	0.5	≥64	<0.5	1	4	<0.5
ECL78	*NDM-1*	*TEM-1*, *SHV-12*	*DHA*	*aac(6′)-Ib-cr*	8	8	>16	>16	>64	>2	>16	8	16	1	4
ECL79	*NDM-1*	*TEM-1*, *SHV-12*	*ACC*	*aac(6′)-Ib-cr*	512	32	≥64	≥64	≥128	0.5	≥64	4	1	2	<0.5
ECL112	*NDM-1*	*TEM-1*	*—*	*aac(6′)-Ib-cr*	4	4	>32	>32	64	>2	16	32	>512	1	<0.5
*E. coli* transconjugant strains															
66TC	*NDM-1*	*TEM-1*	—	—	16	16	16	≥64	≥128	<0.25	≥64	<0.5	<0.5	1	1
67TC	*NDM-1*	*TEM-1*	—	—	4	8	16	≥64	64	<0.25	≥64	<0.5	1	2	1
68TC	*NDM-1*	*TEM-1*	*DHA*	*qnrS*	32	16	32	≥64	≥128	≥4	≥64	4	1	1	1
72TC	*NDM-1*	*TEM-1*	—	—	64	32	16	≥64	≥128	<0.25	≥64	4	<0.5	2	8
73TC	*NDM-1*	*TEM-1*	—	—	64	64	16	≥64	≥128	<0.25	≥64	2	1	2	8
75TC	*NDM-1*	*TEM-1*	—	—	16	16	16	≥64	≥128	<0.25	≥64	<0.5	1	4	1
76TC	*NDM-1*	*TEM-1*	—	—	4	16	32	≥64	≥128	<0.25	≥64	<0.5	<0.5	4	1
78TC	*NDM-1*	*TEM-1*	—	*aac(6′)-Ib-cr*	8	2	16	≥64	≥128	<0.25	≤1	4	8	1	1
79TC	*NDM-1*	*TEM-1*	—	—	16	16	16	≥64	≥128	<0.25	≥64	<0.5	<0.5	1	1
112TC	*NDM-1*	*TEM-1*	—	*aac(6′)-Ib-cr*	2	1	16	≥64	64	<0.25	≥64	8	256	<0.5	<0.5
*EC600*	—	—	—	—	0.5	<0.5	≤1	≤1	≤4	<0.5	≤1	<0.5	≤2	<0.5	<0.5

### Phenotype and genotype analysis

All strains were positive for mCIM and eCIM. PCR assay confirmed that all of the CREL isolates possessed *bla*_NDM−1_ while other carbapenemase genes were not detected. Besides the production of carbapenemase, all of the CREL isolates were positive for both ESBL and AmpC genes. The detection rate of *bla*_TEM_ (100%) was the highest, followed by *bla*_SHV_ (80%) and *bla*_ACC_ (80%), while the detection rate of *bla*_DHA_ was only 20%. Additionally, fluoroquinolone-related genes were also detected to a certain extent, *aac-(6′)-Ib-cr* (100%) was present in all samples, the detection rate of *qnrS* was 10%, and the remaining genes were not detected. It is worth noting that two strains (ECL68 and ECL78) also detected the *mcr-9* gene associated with colistin resistance (**Table 2**).

### Molecular epidemiology of NDM-1-producing CREL isolates

**Figure 1:**
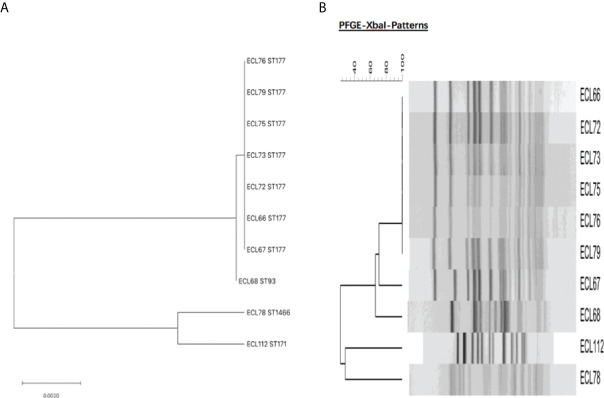
**(A)** Molecular phylogenetic analysis by MLST of NDM-1-producing CREL isolates. **(B)** Dendrogram of the PFGE profiles of NDM-1-producing CREL isolates.

**Figure 2:**
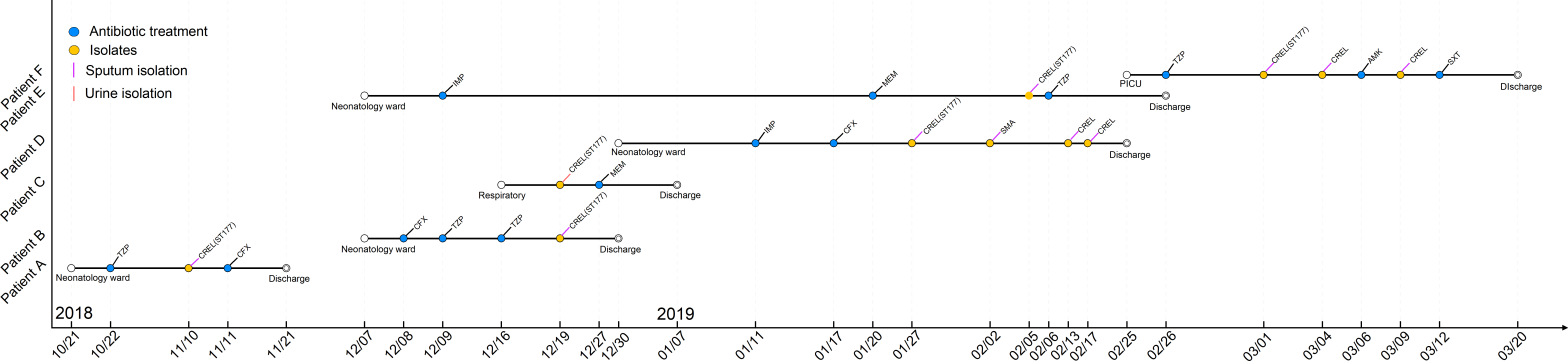
Timeline of the NDM-1-producing CREL ST177 outbreak cases.

A total of four sequence types were detected in the 10 NDM-1-producing CRELs. ST177 was the most common (70%, 7/10), followed by ST93 (10%, 1/10), ST171 (10%, 1/10), and a new sequence type, ST1466 (10%, 1/10). The phylogenetic tree showed four phylogenies of these isolates (**Figure 1A**). It is noteworthy that ST177 was highly homologous to the high-risk clone ST93. By PFGE analyses, these isolates were grouped into A (isolate nos. 66, 72, 73, 75,76,79), B (isolate no. 67), C (isolate no. 68), D (isolate no. 112), and E (isolate no. 78) clusters. Consistent with MLST results, most strains belonging to the same ST were highly similar in their PFGE band patterns (**Figure 1B**). Six isolates belonged to the same clone ST177 and showed an identical PFGE pattern, indicating that outbreak of this ST has occurred in our hospital. The timeline of the outbreak case is depicted in **Figure 2**.

### Plasmid conjugation test

PCR results showed that all of the tranconjugations were positive for the *bla*_NDM-1_ gene, indicating that *bla*_NDM-1_-possessing plasmids from 10 donor strains were successfully transferred into recipient *E. coli EC600*. Some resistance genes were also detected in transconjugants including *mcr-9*, *bla*_TEM_, bla_DHA_, *aac-(6′)-Ib-cr*, and *qnrS*. However, *bla*_SHV_ and *bla*_ACC_ were not detected in tranconjugations. All of the transconjugants showed multidrug resistance phenotypes, which were similar to those of the donor strain. Notably, the degree of resistance (MIC value) and the resistance rate of tranconjugations to some antibiotics were lower than those of donor strains. The results of the drug sensitivity test and resistance gene are summarized in **Table 2**.

### Overview of WGS

**Figure 3:**
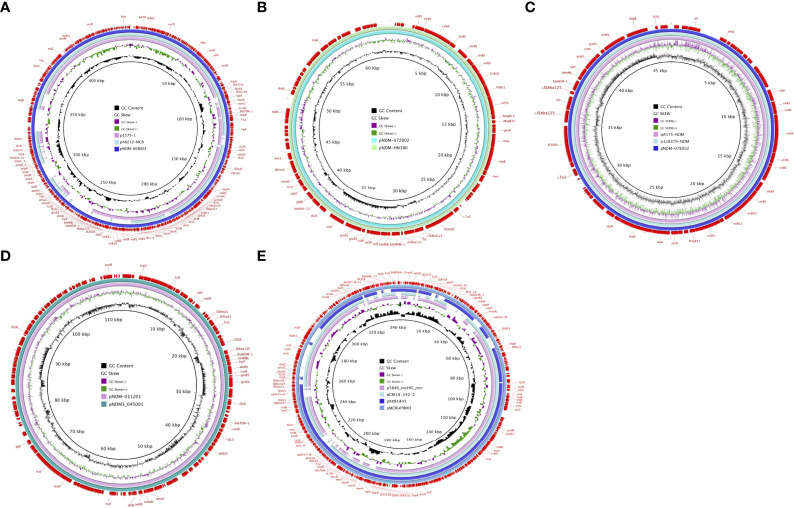
Schematic maps of plasmids harboring *bla*_NDM-1_ and *mcr-9*. **(A)** Circular alignments of the plasmids pNDM-068001 , p1575-1 (accession no. CP068288), and p48212-MCR (accession no. CP059413). **(B)** Map of the plasmids pNDM-072002 and pNDM-HN380 (accession no. NC_019162). **(C)** Map of the plasmids pNDM-078002, pA575-NDM (accession no. MH917283), and p128379-NDM (accession no. MF344560). **(D)** Map of the plasmids pNDM-011201 and pNDM1-045001 (accession no. CP043383). **(E)** Circular alignments of pMCR-078001 sequences with homologous mcr-carrying contigs p3846-IncHI2-mcr (accession no. CP052871), pCM18-242-2 (accession no. CP050507), and pXXB1403 (accession no. CP059887). Genes and open reading frames (ORFs) are labeled in the outermost circle. Truncated genes are indicated by Δ.

High-throughput sequencing with genomic DNA of the ECL 68, 72, 78, and 112 isolates generated six circular sequences of plasmids, of which four carried *bla_NDM-1_* and two carried multidrug-resistant plasmids. pNDM-068001, carried by ECL68, was 444,489 bp in length, with average G+C contents of 47.22%, and contained 490 predicted coding sequences (CDSs). Strain ECL72 harbored two plasmids named pMDR-072001 (175,647 bp) and pNDM-072002 (62,851 bp) with average G+C contents of 48.6% and 48.3%, respectively. Strain ECL78 also harbored two plasmids. pMCR-078001 and pNDM-078002 were 342,942 and 46,352 bp in length, with average G+C contents of 48.56% and 48.53%, respectively. pNDM-011201, carried by ECL112, is a 112,413-bp circular plasmid with an average G+C content of 53.19% and has 120 predicted CDSs. Consistent with multidrug resistance phenotype, these strains harbored one or more plasmid carrying multiple genes mediating resistance to quinolone (*qnrS1*, *qnrB4*, and *qepA*), aminoglycosides [*aac(6’)-IIc*, *aph(3’’)-Ib*, *aac(6’)-Ib-cr*, and *rmtB*], β-lactams (*bla*_NDM-1_, *bla*_TEM−1B_, *bla*_DHA-1_, and *bla*_SHV-12_), bleomycin *ble*_MBL_, trimethoprims (*dfrA* and *sul1*), colistin (*mcr-9*), and MLS—macrolide [*mph(A*)] and tetracycline [*tet(D)*]. All six plasmids encoded plasmid replication (*repAB*), stability (*parABM*), and transfer (*tra* and *virB*) functions. The mobility of these plasmids was confirmed by conjugation assay. The major features of plasmid and schematic maps are summarized in **Table 3** and **Figures 3A–E**.

**Table 3:** The major features of plasmids.

Isolates	Plasmid’s name	Plasmid type	Size (bp)	GC content	Antibiotic resistance genes
ECL68	pNDM-068001	IncHI2/IncN	444,489	47.22%	*bla*_NDM-1_, *ble*_MBL_, *bla*_DHA-_ *_1_*, *bla*_SHV-12_, *tet(D)*, *aph(3’’)-Ib*, *qnrB4*, *qnrS1*, *mcr-9*, *bla*_TEM-1B_, *aac(6’)-IIc*
ECL72	pMDR-072001	Unclassified	175,647	48.60%	*dfrA12, sul1, aph(3’)-Ia, mph(A), bla*_TEM-1B_ *, qacE*
ECL72	pNDM-072002	IncX3	62,851	48.31%	*bla*_NDM-1_, *ble*_MBL_, *bla*_SHV-12_
ECL-78	pMCR-078001	IncHI2	342,942	48.56%	*bla*_TEM-1B_, *bla*_DHA-1_, *bla*_SHV-12_, *aac(6’)-Ib-cr*, *mcr-9*
ECL-78	pNDM-078002	IncX3	46,352	48.53%	*bla*_NDM-1_, *ble*_MBL_, *tet(D)*, *dfrA19*, *sul1*
ECL112	pNDM-011201	Unclassified	112,413	53.19%	*bla*_NDM-1_, *ble*_MBL,_ *bla*_TEM-1B_, *rmtB*

### Analysis of the genetic environment of NDM-1

**Figure 4:**
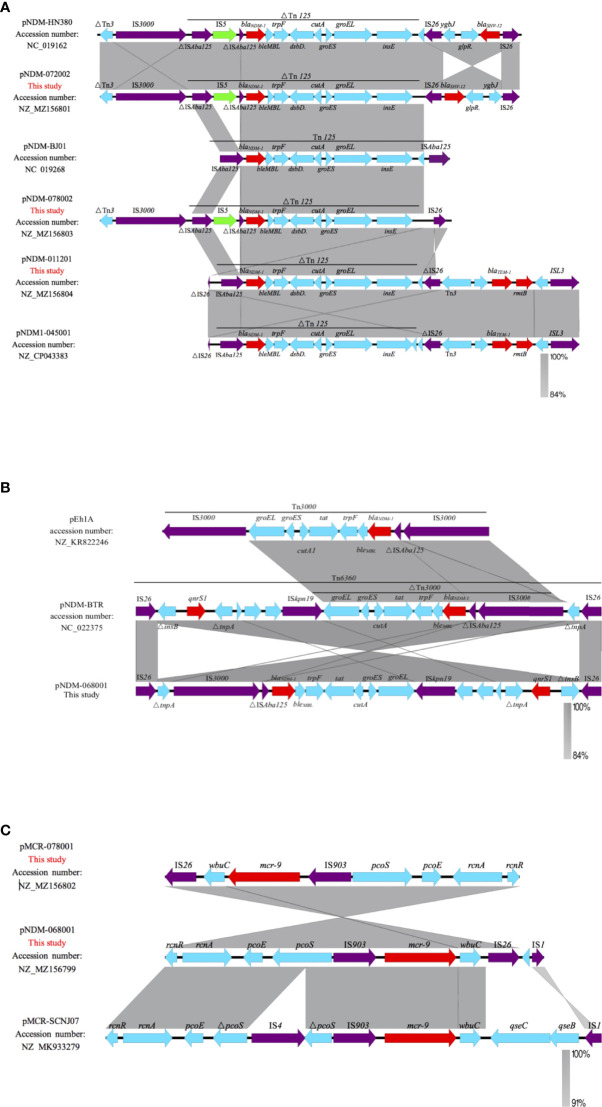
Linearized analyses for genetic environment of *bla*_NDM-1_ and *mcr-9*. **(A)** *bla*_NDM-1_ harbored by pNDM-HN380, pNDM-072002, pNDM-BJ01, pNDM-078002, pNDM-011201, and pNDM-045001. **(B)** *bla*_NDM-1_harbored by pNDM-068001, pEh1A, and pNDM-BTR. **(C)** *mcr-9* harbored by pMCR-078001, pNDM-068001, and pMCR-SCNJ07.

pNDM-072002 and pNDM-078002 are IncX3-type plasmids, which are different from the prototypical Tn*125*. pNDM-072002 forms a derivative of Tn*125* (△Tn*125*). The IS*Aba125* located upstream has an IS*5* insertion, and another IS*Aba125* downstream is lost and replaced by a composite transposon that contains *bla*_SHV-12_ *, ygbJ*, and *glpR* and two copies of IS*26* in flanks. Similarly, pNDM-078002 also shows the △Tn*125*; however, the “IS*26*-*bla*_SHV-12_ composite transposon” is missing, and only one side of IS*26* is retained. Different from the insertion of IS*5*, pNDM-011201 belonged to the unclassified type and formed a ΔTn*125* truncated by IS*26*. Similarly, different from the “IS*26*-*bla*_SHV-12_ composite transposon” structure like pNDM-072002, pNDM1-011201 forms a transposition structure dominated by the *bla*_TEM-1_ drug-resistant gene, which is specifically expressed as “IS*26*-Tn*3*-*bla*_TEM-1_-*rmtB*-IS*L3*” (**Figure 4A**). pNDM-068001 belongs to IncHI2/IncN type plasmid and composed of a transposon named Tn*6360* rather than Tn*125*. The former consists of IS*26*, △Tn*3000*, IS*kpn19*, and △*tnpA*, *qnrS1*, IS*26*. The *bla*_NDM−1_ gene is located in △Tn*3000* between a truncated IS*Aba125* and the *ble_MBL_* gene (**Figure 4B**).

### Analysis of the genetic environment of *mcr-9*

The genetic environments of *mcr-9* in the pNDM-068001 and MCR-078001 were highly similar; *mcr-9* was located in an ~8-kb region surrounded by two insertion sequences IS*903* and IS*26*. The region upstream of *mcr-9* included the conserved gene structure, *rcnR*-*pcoS*-*pcoE*-IS*903*, and *wbuC* was located downstream of *mcr-9*, but lacks the two-component *qseCB* system. A similar structure has been reported in some IncHI2 plasmids such as p58011 (Accession no. CP049309), pN58631 (Accession no. CP049307), and pN18S2238 (Accession no. CP049312). Compared with *E. hormaechei* strain SCNJ07 coharboring *bla*_NDM-1_ and *mcr-9*, pNDM-068001 has a completed *pcoS*, while SCNJ07 was interrupted by IS*4*. The *mcr-9* was surrounded by two insertion sequences, IS*903* and IS*1*; these elements may play a crucial role in transferring mcr-9 (**Figure 4C**).

## Discussion

Increasing antimicrobial resistance is a global emergency that is associated with adverse outcomes for infectious diseases (Uechi et al., 2019). The *bla*_NDM-1_-carrying bacteria conferred resistance to most β-lactam antibiotics, and it is a severe challenge for clinical anti-infection treatment. Previous studies have certified that CREL caused serious infections and resulted in prolonged hospital stay and increased mortality rates (da Silva et al., 2018).

Tigecycline and colistin were the last-resort antibiotics for the treatment of infections caused by multidrug-resistant bacteria including CREL (Doi, 2019). The main mechanism for reduced susceptibility to tigecycline is the upregulation of efflux pumps or mutations. In addition to this, tigecycline-resistant gene, *tet(X)*, is another important mechanism (Li et al., 2020). Meanwhile, with the emergence of colistin-resistant genes, *mcr*, the treatment of CREL infection has become increasingly difficult (Lin et al., 2020). The co-production of multi-resistance genes leads to multidrug resistance, which is a serious threat to public health. Recent studies proposed that plasmid was the crucial vectors for the horizontal transfer of resistance genes (Xiang et al., 2020). In this study, *bla*_NDM-1_-possessing plasmids were successfully transferred into recipient *E. coli EC600*. Notably, the MIC value of most transconjugates for meropenem, imipenem, levofloxacin, and amikacin was less than donor strains, suggesting that the antibiotic resistance degree relied on multiple mechanisms such as changes in bacterial outer membrane proteins. Besides mobile genetic elements, clonal spread was another key factor associated with the prevalence of carbapenem-resistant *Enterobacteriaceae*.

Previous literatures have demonstrated that ST120, ST51, and ST88 NDM-1-producing CREL are widespread in China (Liu et al., 2015). In our study, ST177 was the predominant epidemic type. Interestingly, only one of the seven alleles was different between ST177 and high-risk clones, ST93, indicating that they are closely related to each other. Previous epidemiological survey of CREL in 11 cities showed that ST93 has widely spread in China (Jin et al., 2018), but it has not been reported before in our region. Therefore, epidemiological surveillance measures were necessary to control this pathogen’s further spread. Homology analysis showed that six isolates belonged to ST177 and exhibited an identical PFGE pattern, suggesting that a small-scale outbreak occurred in our hospital. Strain ECL79, retrieved from the neonatology ward, may be the index case in this event. The outbreak strains were mainly distributed in the neonatology ward. However, ECL66 and ECL75, isolated from PICU and the respiratory ward, respectively, showed highly genetic homologous strains isolated from the neonatology ward. Notably, ECL67, which belonged to ST177 and isolated from the pediatric ward, was assigned to a different cluster by PFGE analysis, indicating that this case was unrelated to outbreak. Outbreaks of NDM-1-producing *E. cloacae complex* ST74 and ST88 have been reported in Yunnan and Chongqing, respectively (Du et al., 2017; Jia et al., 2018). As far as we know, this is the first report of ST177 NDM-1-producing CREL outbreak in China. The outbreak of this sequence type may provide a new insight into this pathogen and should be taken seriously.

In this work, plasmid analysis revealed that pNDM-072002 and pNDM-078002 belonged to the IncX3 group, which have a broad host range and self-conjugation. pNDM-072002 is almost identical to the plasmid pNDM-HN380 (99% identity and 99.9% coverage) from *K. pneumoniae* from Hongkong in 2020, and there is mainly a ~3.5-kb inversion difference between the two plasmids. pNDM-078002 is highly similar to the plasmid pA575-NDM (99% identity and 99.9% coverage) from *K. pneumoniae* from Beijing, China, in 2020 and p128379-NDM (99% identity and 99.9% coverage) from *Enterobacter hormaechei* from Beijing, China, in 2020. Meanwhile, the *bla*_NDM-1_-containing plasmid IncX3 was commonly detected in *Enterobacteriaceae*, such as p112298-NDM (Accession no. KP987216) from *Citrobacter freundii* from Beijing and pNDM-SCCRK18-72 (Accession no. MN565271) from *Escherichia coli* from Chengdu. Our findings are consistent with previous results that show that IncX3-type plasmids are most common in *Enterobacteriaceae* obtained from various regions in China (Li et al., 2020). The prototype Tn*125*, carried by plasmid pNDM-BJ01 (Accession no. NC_019268), was isolated from *Acinetobacter lwoffii* and sequenced as IS*Aba125*-*bla*_NDM-1_-*ble*_MBL_-*trpF*-*dsbD*-*cutA*-*groES*-*groEL*-*insE*-IS*Aba125* (Feng et al., 2015). In our study, *bla*_NDM-1_-carrying plasmids belong to the transposon Tn*125* variant except for pNDM-068001. Different from prototype Tn*125*, the upstream IS*Aba125* is interrupted by IS*5*, and the downstream IS*Aba125* is replaced by IS*26* in pNDM-078002 and pNDM-072002. pNDM-011201 has no IS*5* insertion upstream, but IS*Aba125* is truncated by IS*26*. This variant was also found in the pNDM1-045001 (NZ_CP043383) plasmid reported in Chengdu, Sichuan Province, China. Interestingly, the MDR region in pNDM-068001 is composed of Tn*6360* rather than Tn*125*. The former consists of △Tn*3000*, Tn*6292*, IS*kpn19*, △*tnpA*, and IS*26* and was first identified in pNDM-BTR (NC_022375) (Chen et al., 2020). Thus, Tn*3000* and Tn*629* may be reorganized and then Tn*6360* is created.

The prevalence of *mcr-9* is not clear because many isolates carry *mcr-9* genes but still displayed colistin-susceptible phenotypes (Chavda et al., 2019). The *mcr-9* gene, which may be spreading undetected in the world, was first identified in *Salmonella typhimurium* in 2019 (Carroll et al., 2019). Notably, we found ECL68 and ECL78 co-carrying *bla*_NDM-1_ and *mcr-9*. According to accessible literature, the co-existence of *mcr-9* and carbapenems are not rare. Soliman et al. (2020) and Yuan et al. (2019) reported the co-existence of *mcr-9* and *bla_VIM-4_* or *bla_NDM-1_* in the *E. hormaechei* isolate, respectively (Yuan et al., 2019; Soliman et al., 2020). Kananizadeh et al. (2020) revealed co-carrying of *bla*_IMP-1_ and *mcr-9* in a clinic *E. cloacae complex* strain (Kananizadeh et al., 2020). These findings highlight that active monitoring strategies should be implemented. Tyson et al. (2020) reported sensor *qseC* and response regulator *qseB*, also known as the two-component system *qseCB*, to be involved in the regulation of *mcr-9* (Tyson et al., 2020). In the present study, IS*903* was located upstream of *mcr-9*, *wbuC* was located downstream, followed by IS*26*, and the two-component system *qseCB* was absent. Strains ECL68 and ECL78 carry mcr-9 but remain susceptible to colistin probably due to the absence of the *qseC/qseB* system. Considering that the prevalence of mcr-9 is not unclear, active surveillance is necessary to control the further spread.

In conclusion, we first reported a nosocomial outbreak of NDM-1-carrying *E. cloacae complex* ST177 in China. Prevention strategies should be implemented to prevent NDM-1-producing bacteria transmission. The potential role of mcr-9 in colistin resistance needs further investigation.

## Data availability statement

The datasets presented in this study can be found in online repositories. The names of the repository/repositories and accession number(s) can be found below: https://www.ncbi.nlm.nih.gov/genbank/, MZ156799 https://www.ncbi.nlm.nih.gov/genbank/, MZ156800 https://www.ncbi.nlm.nih.gov/genbank/, MZ156801 https://www.ncbi.nlm.nih.gov/genbank/, MZ156802 https://www.ncbi.nlm.nih.gov/genbank/, MZ156803 https://www.ncbi.nlm.nih.gov/genbank/, MZ156804.

## Ethics statement

Ethical review and approval was not required for the study of human participants in accordance with the local legislation and institutional requirements. Written informed consent from the patients/participants or patients/participants legal guardian/next of kin was not required to participate in this study in accordance with the national legislation and the institutional requirements.

## Author contributions

All authors contributed to the article and approved the submitted version. XZ designed the study. KH and JSZ wrote this paper. JSZ, JBZ, WL, and JL conducted the experiments. KH, LZ, and JW analyzed the data. All authors contributed to the article and approved the submitted version.

## Funding

This work was supported by the General Projects of Chongqing Natural Science Foundation (cstc2020jcyj-msxm0067), the Yongchuan Natural Science Foundation (2021yc-jckx20053), and the Talent Introduction Project of Yongchuan Hospital of Chongqing Medical University (YJYJ202005 and YJYJ202004).

## Acknowledgments

We thank the curators of the Institute Pasteur MLST system (Paris, France) for approved novel alleles and profiles at http://bigsdb.pasteur.fr.

## Conflict of interest

The authors declare that the research was conducted in the absence of any commercial or financial relationships that could be construed as a potential conflict of interest.

## Publisher’s note

All claims expressed in this article are solely those of the authors and do not necessarily represent those of their affiliated organizations, or those of the publisher, the editors and the reviewers. Any product that may be evaluated in this article, or claim that may be made by its manufacturer, is not guaranteed or endorsed by the publisher.
